# Dietary Green Tea Enhances the Growth, Antioxidant Capacity, and Abundance of Beneficial Intestinal Flora of Grass Carp (*Ctenopharyngodon idellus*)

**DOI:** 10.3390/ani15243595

**Published:** 2025-12-15

**Authors:** Yuyan Li, Ruiyi Yang, Shaoyu Zhu, Cong Wang, Rui Wang, Dingding Yue, Yuning Wang, Yanou Yang

**Affiliations:** 1 College of Animal Science and Technology, Anhui Agricultural University, Hefei 230036, China; liyuyan@stu.ahau.edu.cn (Y.L.); zhushaoyu979@outlook.com (S.Z.); yangahnd2@126.com (C.W.); wangruikeshi@163.com (R.W.); yuedingding@lsstjt.com (D.Y.); wangyuning202509@163.com (Y.W.); 2 School of Cellular and Molecular Medicine, University of Bristol, Bristol BS8 1QU, UK; bz22972@bristol.ac.uk

**Keywords:** grass carp (*Ctenopharyngodon idellus*), green tea, growth, metabolic enzymes, antioxidation, intestinal flora

## Abstract

Tea is a widely consumed natural herbal plant boasting diverse beneficial properties, yet its effects vary significantly with different dosages. To study the effects of green tea dosage on the growth, feed utilization, antioxidant capacity, and diversity and abundance of intestinal flora of grass carp, 0 (control group), 500, 1000, 2000, 4000, and 8000 mg kg^−1^ of green tea were added to the basal diet of grass carp for 8 weeks. The results showed that the grass carp in the 1000 mg kg^−1^ group had a significantly higher feed efficiency ratio (FER) than in the control group (an increase of 5%), which may be associated with the increasing glycolysis and intestinal protease activity. In the 8000 mg kg^−1^ group, grass carp had a significantly lower FER and growth performance, which might be related to a decrease in gluconeogenesis. Green tea improved the antioxidant capacity of grass carp, with the 1000 mg kg^−1^ group exhibiting the best efficacy. At this dosage, green tea significantly decreased the abundance of harmful intestinal bacteria and increased that of beneficial bacteria. The findings indicate that 1000 mg kg^−1^ of green tea enhances grass carp’s growth performance, antioxidant capacity, and intestinal immunity. This study clarified the mechanism by which green tea improves the growth performance of grass carp through enhancing antioxidant capacity, metabolic regulation, and microbiota remodeling, providing strong theoretical support for the application of green tea in aquaculture. In the future, more research on the mechanisms underlying the effects of green tea on grass carp is necessary.

## Simple Summary

Green tea has many beneficial functions and has been studied in the breeding of livestock and poultry. Therefore, it can be considered for use in aquaculture. Grass carp is an economically important farmed fish in China, which grows rapidly but is prone to diseases. Therefore, in this study, different amounts of green tea were added to the diet of grass carp as part of an 8-week feeding experiment. The results showed that an appropriate amount of green tea can significantly improve the feed utilization and antioxidant capacity of grass carp, leading to a significant decrease in the abundance of harmful intestinal bacteria and a significant increase in that of beneficial bacteria, thereby enhancing intestinal immunity. This study aims to improve the healthy aquaculture level of grass carp.

## 1. Introduction

Grass carp (*Ctenopharyngodon idellus*) is an important freshwater-farmed herbivorous fish in China due to its rapid growth, tasty meat, and low price per unit of production [1]. China’s grass carp production was above six million tons, accounting for 22% of China’s freshwater fish production [2]. Currently, the high-density culture of grass carp is becoming a common practice to increase yield per unit water volume [3], leading to stronger stress responses and more diseases [4], such as bacterial infections [5] and parasitic infections [6]. In response, more fish medicine has been used, causing more water and environmental pollution [7]. Vaccination is used to enhance the immunity of grass carp, but it is troublesome to administer and costly [8]. Genetic engineering is also used to improve the immunity of fish, but its large-scale application takes a long time [9]. Therefore, developing new diet ingredients that can enhance immunity and antioxidant capacity in fish is a feasible idea. Due to their relative safety and fewer side effects, herbal plants can be considered as dietary additives [9].

Tea is a widely used natural herbal plant with numerous beneficial effects on human health, such as antioxidation, induction of metabolic enzymes, inhibition of cell proliferation, and regulation of intestinal flora [10]. In recent years, the application of tea extracts, such as tea polyphenols (TPs), has been reported in aquaculture [11,12,13,14]. Studies have shown that TPs can enhance the antioxidant capacity and promote the growth of aquatic animals [15,16,17]. Among various types of tea, green tea is a non-fermented tea with a high content of TPs [18,19]. During the processing of green tea, a considerable amount of tea fragments are produced [20,21], which are inexpensive and can be used in fish diets. In addition to TPs, green tea contains theanine, alkaloids, polysaccharides, etc., and the functions of green tea may differ from those of TPs [10]; therefore, more research on the effects of green tea in fish is needed. Studies have shown that 100 mg kg^−1^ of green tea (by dry leaf weight; the same applies below) in the diet can significantly increase the activities of superoxide dismutase (SOD) and glutathione peroxidase (GPx) in the serum of rainbow trout (*Oncorhynchus mykiss*), and significantly reduce the content of malondialdehyde (MDA), whereas 20 mg kg^−1^ and 500 mg kg^−1^ of green tea have no significant effect on these indicators [18]. For channel catfish (*Ictalurus punctatus*) [22] and Yellow River carp (*Cyprinus carpio*) [23], different doses of green tea have different effects on antioxidant indicators. Green tea has been shown to influence fish growth performance and feed utilization efficiency. For Nile tilapia (*Oreochromis niloticus*), dietary supplementation with 250 and 500 mg kg^−1^ of green tea results in a significantly higher final body weight and feed efficiency ratio (FER) [24]; for hybrid tilapia (*Oreochromis niloticus* × *O. aureus*), 8000 and 16,000 mg kg^−1^ of green tea achieved the same significant effect [25]; For grass carp [26] and juvenile black rockfish (*Sebastes schlegeli*) [27], which both show significantly improved weight gain and FER, the optimal supplementation levels differ considerably. In the aforementioned studies, other supplementation doses for each fish species either had no significant effect on SGR and FCE or significantly reduced these two parameters. These studies indicate that green tea influences both the antioxidant capacity and growth performance of fish, and the effect is highly dependent on the amount of additive and variations between fish species. As mentioned above, grass carp is a major farmed fish species in China. However, research on the effects of green tea on grass carp is presently insufficient, and the mechanisms underlying its influence on growth remain unclear. Therefore, utilizing green tea from Anhui Province, China, we experimentally explored the effects of green tea on the growth, antioxidant capacity, and intestinal flora of grass carp, in order to expand the application of tea leaves, improve the diet formula, and enhance the farmed efficiency of grass carp.

## 2. Materials and Methods

### 2.1. Fish and Experimental Diets

Grass carp were obtained from Chaohu Farm (Hefei, Anhui Province, China), and the Experimental Fish Purchase Informed Consent was also obtained. The green tea variety was Huangshan Maofeng, grown in Huangshan City, Anhui Province, China. The tea was picked, and various indicators were measured at College of Tea Science of Anhui Agricultural University, with the determination methods referring to respective standards [28,29,30,31,32]. The main components of the green tea were water extract (52.38%), TPs (21.32%), free amino acids (3.44%), theanine 10.25 mg g^−1^, and caffeine 40.05 mg g^−1^ (dry weight). Before the experiment, the fish were temporarily reared in a recirculating aquaculture system for 2 weeks and fed a basic diet (Table 1). Based on our previous preliminary test and reference [26], 0, 500, 1000, 2000, 4000, and 8000 mg kg^−1^ of green tea powder were added to the basic diet and recorded as the G0 (Control), G500, G1000, G2000, G4000, and G8000 groups. All kinds of materials were ground into powder through a 60 mesh-graded sieve and made into 2 mm particles with a small-scale pellet feed machine (SLP-45, Institute of Fishery Machinery and Instruments, Chinese Academy of Fishery Sciences, Shanghai, China), dried in an air-dry oven at 60 °C, sieved, and sealed in a plastic bag at 4 °C.

### 2.2. Experimental Procedure and Feeding Management

The experimental system was an indoor recirculating aquaculture system containing 18 tanks (60 cm × 60 cm × 60 cm; volume, 180 L). The central water purification system utilized a combination of zeolite, activated carbon, bacteriophage balls, coral stone, and cotton filter media (filter material from Beishankou Filter Materials Co., Ltd. of Gongyi City, Henan Province, China) to treat the water, which is sourced from aerated tap water. There was an air stone in each tank, and the tanks were oxygenated with a root blower connected to a ventilation pipe. During the trial period, the circulating water volume of each tank was 2.50 L min^−1^, dissolved oxygen was at least 7.0 ± 0.5 mg L^−1^, ammonia nitrogen was less than 0.10 mg L^−1^, pH was about 7.5, water temperature was 25 ± 1.0 °C, and the light time was 12 h, from 8:00 to 20:00.

The fish were fasted for 24 h, weighed, and recorded as the initial body weight (Table 2) before feeding. They were then distributed randomly into each tank, with 30 fish in each. The test consisted of 6 groups, each containing 3 tanks, and each tank was considered one experimental unit for statistical analysis. The fish were fed to apparent satiation twice a day (at 9:00 and 15:00) for 8 weeks, and the residual diet was recovered after 1 h at each feeding and dried in an air-dry oven at 65 °C. The amount of residual diet was corrected by feed dissolution rate, which was determined by randomly placing one weighed portion of feed in each of the three fishless tanks, recovering it after 1 h, drying it to constant weight, and weighing it.

### 2.3. Sample Collection

During the trial, no mortalities or behavioral changes occurred. After 8 weeks, the grass carp were starved for 24 h before sampling. A certain amount of water in each tank was drained, the anesthetic MS-222 (Syndel Company, Yichang, China, 200 mg L^−1^) was added, and then the fish were weighed and counted. Seven fish were randomly collected from each tank and measured for body length and weight to calculate condition factor (CF). Fish bodies were placed on ice trays, removing the internal organs, liver, all segments of the intestine, and muscles on the body’s central back (sample area of 1 cm^2^ on each side from below the dorsal fin). The internal organs and liver were weighed to calculate the viscerosomatic index (VSI) and hepatosomatic index (HSI). The liver, intestine, and muscles of each fish were mixed separately and placed in cryopreservation tubes to analyze enzyme activity, glycogen content and intestinal flora. Another seven fish were randomly selected from each tank and used as one sample for the analysis of whole fish body biochemical composition. All samples were collected and stored at −80 °C.

### 2.4. Determination of Biochemical and Physiological Indices

The contents of moisture, crude protein, crude lipid, and ash in the diet and whole fish were determined by the Association of Official Analytical Chemists [33]. The moisture content was determined using the drying method (the sample was dried to constant weight at 105 °C), the crude protein content using Kjeldahl nitrogen determination, the crude lipid content with Soxhlet extraction, and the ash content using Muffle furnace burning at 550 °C. The diet energy was measured using a bomb calorimeter (IKA, C 6000, Staufen, Germany).

The physiological indices included the activities of intestinal trypsin, lipase, and amylase; the activities of liver hexokinase (HK), pyruvate kinase (PK), lactic dehydrogenase (LDH), succinate dehydrogenase (SDH), phosphoenolpyruvate carboxykinase (PEPCK), glucose-6-phosphatase (G-6-Pase), alanine aminotransferase (ALT), aspartate aminotransferase (AST), lipoprotein lipase (LPL), hepatic lipase (HL), total lipase (TL), superoxide dismutase (SOD), catalase (CAT), and glutathione peroxidase (GPx); the contents of malondialdehyde (MDA) and glutathione (GSH); the total antioxidant capacity (T-AOC); and the contents of hepatic glycogen (HG) and muscle glycogen (MG). These indicators were measured using a kit from the Nanjing Jiancheng Institute of Bioengineering (Nanjing, China). For specific methods, please refer to the relevant kits.

### 2.5. Sequencing of Intestinal Flora

The control group and the group with the best feed utilization were used as the sequencing samples. Total bacterial genomic DNA was extracted using a Qiagen kit (New York, NY, USA), according to the manufacturer’s instructions. The V1–V9 hypervariable region of the 16S rRNA gene was PCR amplified using the forward primer 27F (5’-AGRGTTTGATYNTGGCTCAG-3’) and the reverse primer 1492R (5’-TASGGHTACCTTGTTASGACTT-3’). Amplification products were then purified and recovered using agarose gel electrophoresis (1.0%). Finally, library construction and sequencing analysis were conducted by Beijing Biomarker Technologies Co., Ltd. (Beijing, China). Bioinformatics analysis was performed on the Biomarker Biocloud platform (https://www.biocloud.net/). The qualified sequences with more than 97% similarity thresholds were allocated to one operational taxonomic unit (OTU) using USEARCH (version 10.0). Taxonomy was assigned to all OTUs by searching against the Silva databases (https://www.arb-silva.de/). The QIIME2 software (Release 2024.2) was used to perform Alpha diversity analysis [34]. The differential abundance of intestinal flora from different groups was assessed using the linear discriminant analysis effect size (LEfSe) [35].

### 2.6. Calculations and Statistical Analysis

Specific growth rate (SGR, % d^−1^) = 100% × [ln (final body weight) − ln (initial body weight)]/days (1)

Feeding rate (FR, % d^−1^) = 100 × dry feed intake × 2/[(final body weight + initial body weight) × days] (2)

Feed efficiency ratio (FER, %) = 100 × [(final body weight − initial body weight)/feed intake (g)](3)

Condition factor (CF, g cm^−3^) = 100 × (body weight/body length^3^) (4)

Viscerasomatic index (VSI, %) = 10 × (visceral weight/body weight)(5)

Hepatosomatic index (HSI, %) = 100 × (hepatopancreas weight/body weight)(6)

Mesenteric fat index (MFI, %) = 100 × (mesenteric fat weight/body weight)(7)

The initial body weight is IBW, and the final body weight is FBW. The data analyses were conducted using the SPSS 24.0 software. First, the data were tested for normality (Shapiro–Wilk test) and homogeneity of variance (Levene’s test); then, one-way ANOVA was performed to evaluate the impacts of the different doses of green tea on growth performance, metabolism, and antioxidant capacity, followed by Duncan’s multiple comparisons of differences among groups. An unpaired *t*-test was used to compare the differences in α-diversity indices between the control and the treatment groups. LEfSe analysis was performed using the Kruskal–Wallis test and linear discriminant analysis (LDA), with an LDA score threshold > 2.5. The values were statistically significant when *p* < 0.05, and descriptive statistical values are expressed as the mean ± SD in the tables.

## 3. Results

### 3.1. Growth Performance, Feed Utilization, and Body Indices

As shown in Table 2, the G500, G1000, and G2000 groups had FBW and SGR similar to the control group (*p* > 0.05). The G500 and G2000 groups had FER similar to the control group (*p* > 0.05), and the G1000 group showed a significantly higher FER than the control group (*p* < 0.05). These indices decreased significantly in the G4000 and G8000 groups (*p* < 0.05). Compared to the control group, the G500, G1000, and G2000 groups had a similar FR (*p* > 0.05); the G4000 and G8000 groups had a significantly higher FR (*p* < 0.05); the G1000 group was also significantly higher in terms of FER; and the G4000 and G8000 groups had a significantly lower FER (*p* < 0.05). There were no significant differences in CF, VSI, and MFI among all groups (*p* > 0.05).

### 3.2. Whole Body Composition

As shown in Table 3, there were no significant differences in the contents of moisture, crude protein, crude lipid, and ash among all groups (*p* > 0.05).

### 3.3. Digestive Enzyme Activities

As shown in Table 4, the G1000 group exhibited significantly increased trypsin activity compared to the control and G8000 groups (*p* < 0.05), and the G8000 and the other groups showed similar levels to the control group (*p* > 0.05). All groups had similar lipase activities (*p* > 0.05), and all green tea groups had lower amylase activity than the control group, while G1000 and G8000 reached a significant level (*p* < 0.05).

### 3.4. Metabolic Enzyme Activity and Liver Glycogen and Muscle Glycogen Content

As shown in Table 5, all the tea groups had higher HK and PK activities than the control group, with the G500 and G1000 groups showing a significant increase (*p* < 0.05). There was no significant difference in LDH activity between all groups (*p* > 0.05). The G4000 and G8000 groups had significantly lower PEPCK and G-6-Pase activities than the control group (*p* < 0.05). All groups had similar HL and TL activities, and similar MG content (*p* > 0.05). All the tea groups had significantly lower HG than the control group (*p* < 0.05).

### 3.5. Antioxidant Index

As shown in Table 6, the tea groups had significantly higher SOD and T-AOC than the control group (*p* < 0.05). All groups had similar MDA (*p* > 0.05). Compared to the control group, the G500, G1000, and G8000 groups had significantly higher GPx (*p* < 0.05), and the G500 and G1000 groups had significantly higher CAT (*p* < 0.05), while the G8000 group had significantly lower CAT and GSH (*p* < 0.05).

### 3.6. Effects of Green Tea on Intestinal Flora

#### 3.6.1. Richness and Diversity

**Figure 1:**
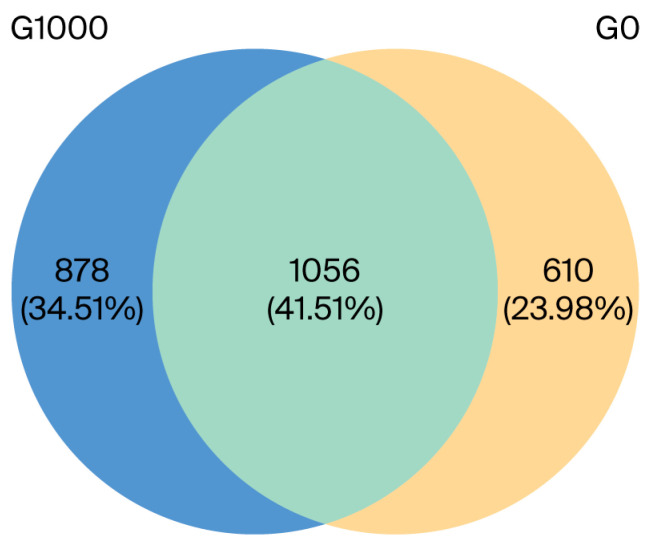
Venn diagram analysis depicting the numbers of shared and unique OTUs of intestinal flora of grass carp (*n* = 4). Note: G0: control group; G1000: treatment group.

**Figure 2:**
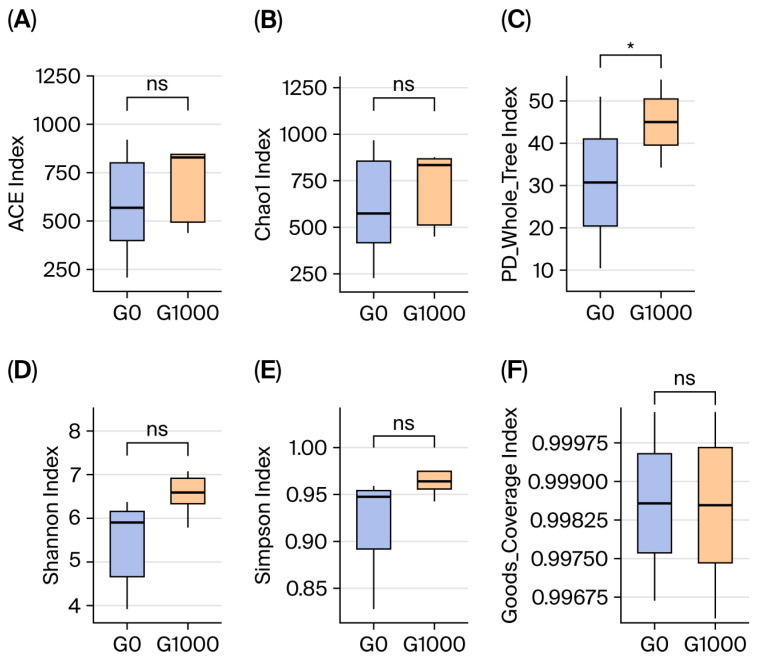
Effect of dietary green tea on intestinal microbiota diversity of grass carp (*n* = 4). Note: (**A**) ACE index; (**B**) Chao 1 index; (**C**) PD_whole_tree index; (**D**) Shannon index; (**E**) Simpson index; (**F**) Goods_Coverage index. G0: control group; G1000: treatment group. *: Significant difference between two groups at *p* < 0.05; ns: no significant difference between two groups at *p* > 0.05.

There were 1056 common OTUs in the G0 and G1000 groups, accounting for 41.51% of the total. The independent OTUs were 610 and 878 in the G0 and G1000 groups, respectively (Figure 1). The diversity and richness of the intestinal flora were assessed using the α-diversity index. The PD_whole_tree was significantly higher in the G1000 group than in the G0 group (*p* < 0.05) (Figure 2C).

#### 3.6.2. Community Composition and Biomarker Analysis

**Figure 3:**
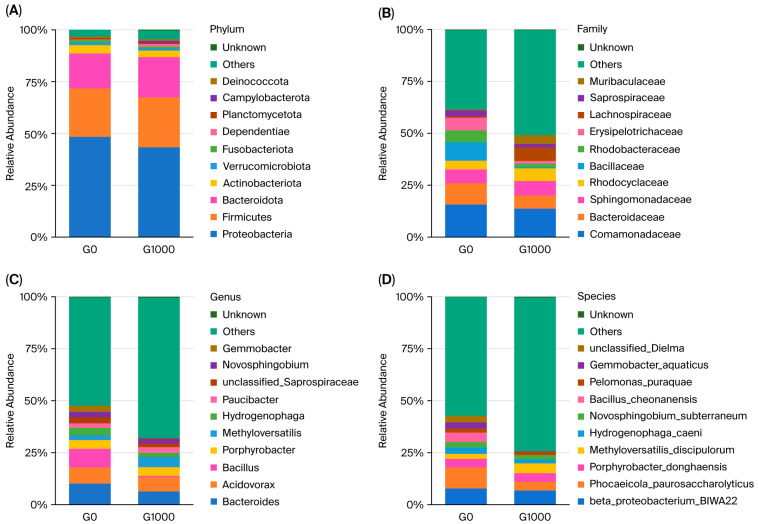
Analysis of dominant flora in the intestine of grass carp (*n* = 4); each bar represents the average relative abundance of the bacteria. Note: (**A**) phylum level; (**B**) family level; (**C**) genus level; (**D**) species level. G0: control group; G1000: treatment group.

A histogram of the relative abundance, containing the top 10 dominant bacteria, was shown at the phylum level for grass carp (Figure 3A). The main dominant bacterial phyla included Proteobacteria, Firmicutes, Bacteroidota, and Actinobacteriota.

A histogram of the relative abundance, containing the top 10 dominant bacteria, was shown at the family level (Figure 3B). The main dominant bacterial families included Comamonadaceae, Bacteroidaceae, Sphingomonadaceae, and Rhodocyclaceae.

A histogram of the relative abundance, containing the top 10 dominant bacteria, was shown at the genus level (Figure 3C). The main dominant bacterial genera included *Bacteroides*, *Acidovorax*, *Bacillus*, and *Porphyrobacter*.

A histogram of the relative abundance, containing the top 10 dominant bacteria, was shown at the species level (Figure 3D). The main dominant bacterial species included *beta_proteobacterium_BIWA22*, *Phocaeicola_paurosaccharolyticus*, *Porphyrobacter_donghaensis*, and *Methyloversatilis_discipulorum*.

**Figure 4:**
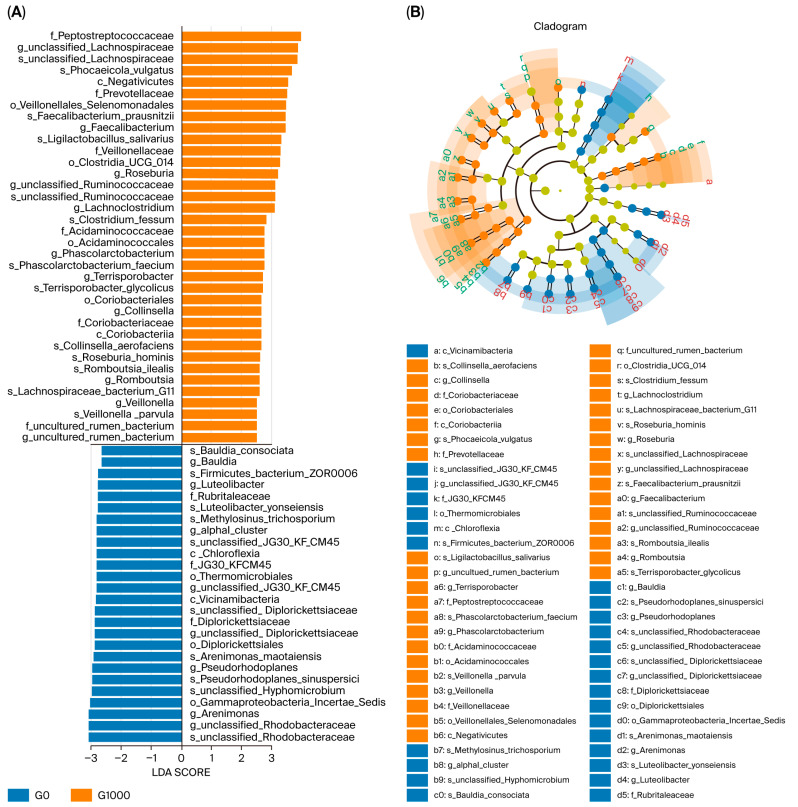
LEfSe analysis of intestinal flora of grass carp (*n* = 4). Note: LDA score (**A**) and taxonomic cladogram (**B**) from LEfSe analysis of 16S sequences (LDA SCORE > 2.5, *p* < 0.05, Kruskal–Wallis test). The rings represent phylum, class, order, family, genus, and species from inside to outside. G0: control group; G1000: treatment group.

The results of the LEfSe analysis with LDA > 2.5 as a threshold are shown in Figure 4. The G0 group had 26 significantly different groups of bacteria, including Bauldia_consociata, Bauldia, Firmicutes_bacterium_Zor0006, *Luteolibacter*, and Rubritaleaceae; the G1000 group had 36 significantly different groups of bacteria, including Peptostreptococcaceae, unclassified_Lachnospiraceae, *Phocaeicola_vulgatus*, Negativicutes, and Prevotellaceae.

## 4. Discussion

### 4.1. Effects on Growth Performance, Feed Utilization, and Digestive and Metabolic Enzymes

For black rockfish, 10,000 mg kg^−1^ and 30,000 mg kg^−1^ of green tea did not significantly affect FER and SGR, while 50,000 mg kg^−1^ of green tea significantly reduced FER and SGR [27]. In a study that involved feeding Nile tilapia with 125, 250, 500, 1000, and 2000 mg kg^−1^ of green tea, only the 250 and 500 mg kg^−1^ groups had a significantly higher FER and SGR than the control group [24]. In this study, the FER and SGR of fish have a similar changing trend to the aforementioned Nile tilapia experiment. The above studies indicate that an appropriate level of green tea can improve feed utilization and growth, while excessive levels exert inhibitory effects. Thus, appropriate dietary green tea supplementation is highly beneficial for reducing feed costs and enhancing aquaculture economic benefits [36]. When feeding hybrid tilapia with diets containing either green tea or TPs, the intestinal amylase activity decreased significantly or markedly [25,37], respectively, which has a similar trend to the results of our study. A possible reason is that amylase contains many hydrophobic amino acids (such as proline, phenylalanine, and tyrosine) [38,39], and the structure of green tea containing non-polar groups such as long carbon chains and aromatic rings has hydrophobic functions and may cause changes in the molecular conformation of amylase, thereby reducing amylase activity [40,41]. The decline in amylase activity may also be associated with green tea tannins; specifically, the catechin compounds in green tea tannins contain multiple phenolic hydroxyl groups (-OH), and these polar groups can bind to amylase molecules via hydrogen bonds and hydrophobic interactions, thereby reducing amylase activity [42]. In addition, green tea tannins can also bind to starch (particularly amylose) to form tannin/starch complexes, thereby inhibiting amylase activity [43]. Reduced amylase activity impairs carbohydrate digestibility and decreases the amount of glucose generated via intestinal decomposition, which may induce a decline in blood glucose levels. Consequently, the liver breaks down stored hepatic glycogen into glucose and releases it into the bloodstream [43], leading to a significant reduction in hepatic glycogen levels in the green tea-supplemented group. In this study, green tea had no significant effect on intestinal lipase activity, which is consistent with Zheng’s report on hybrid tilapia fed with tea residues [25]. However, dietary TPs significantly increased intestinal lipase activity in koi carp (*Cyprinus carpio*) [44], and dietary tea residues had the same effect in largemouth bass [45]. These discrepancies may be ascribed to variations in the dosage of tea-derived supplements or the inherent feeding habits of fish. Grass carp and tilapia are typical herbivorous and omnivorous fish, respectively [25]. The koi carp is omnivorous but prefers animal matter, while the largemouth bass is a typical carnivorous fish; these two species are more adept at utilizing fat [44,45]. More research is needed on how tea affects intestinal lipase activities in fish. The G1000 group had the significantly highest protease activity. Higher protease activity promotes more effective dietary protein utilization, enhancing aquaculture benefits. Simultaneously, reduced fecal protein content alleviates water nitrogen pollution, improving water quality and reducing the incidence of disease [46].

HK and PK are key rate-limiting enzymes in the glycolytic pathway, and their enhanced activities can promote the utilization of carbohydrates, supply intermediate products for biosynthesis, and improve feed utilization [47,48]. Therefore, the increase in FER in the G500 and G1000 groups may be related to the enhancement of glycolysis. The activities of PEPCK and G-6-Pase, as the rate-limiting enzymes of gluconeogenesis, were significantly decreased in the G4000 and G8000 groups. This change directly indicates a decrease in gluconeogenesis. When gluconeogenesis in bony fish is inhibited, the glucose produced by the liver decreases, which cannot meet the energy requirements of the fish, and the fish will shift their energy sources to fat (through β-oxidation) and protein (through amino acid deamination) [48]. Thus, the inhibition of gluconeogenesis tends to cause fish to break down fat and protein for energy supply, since fat and protein are also the core matrices for body tissue development, which might lead to reduced growth [49,50]. For instance, when grass carp overwinter, the AMPK pathway is activated, significantly down-regulating the expression of key genes for gluconeogenesis. Gluconeogenesis is inhibited, leading to the decomposition of fat and protein for energy in the fish’s body and a significant decrease in body weight [51]. The gluconeogenesis ability of mudskips is insufficient, which accelerates the breakdown of muscle protein and fat, leading to rapid weight loss [52]. Therefore, the final body weights in the G4000 and G8000 groups were significantly reduced, which might be related to the decline in gluconeogenesis.

ALT catalyzes the production of glutamic acid, an important amino acid and a precursor for the synthesis of other non-essential amino acids, and the decline in ALT activity may indicate a blockage in protein synthesis [53]; SDH is the only multi-subunit enzyme in the tricarboxylic acid (TCA) cycle that is bound to the inner mitochondrial membrane, and its activity is generally used to determine the operation degree of the TCA cycle. The decrease in SDH activity may lead to the inhibition of the TCA cycle and a reduction in ATP, resulting in growth inhibition [54,55]. In this study, the AST activity in the G8000 group was significantly higher than that in the control and the other green tea groups. AST is mainly present in mitochondria, and increased AST activity usually indicates cell damage or mitochondrial dysfunction [53]. The significantly lowest FER and SGR in the G8000 group might also be related to the significant increase in AST.

### 4.2. Effect on Antioxidant Status

Rainbow trout [18], channel catfish [22], and Yellow River carp [23] had significantly enhanced antioxidant capacity after feeding on green tea, similar to our study, which might be related to the higher content of TPs in green tea. At a dietary TP level of 300 mg kg^−1^, hybrid sturgeon (*Acipenser baerii* ♀ × *A. schrenckii* ♂) showed significantly increased hepatic T-AOC, CAT, SOD, and GSH contents [14]. When dietary TPs were 400 mg kg^−1^, hybrid crucian carp (HCC2) activated the Nrf2/Keap1 and PPARα pathways, with significantly elevated serum SOD, CAT, and GSH levels, thereby enhancing the fish’s antioxidant capacity [12]. TPs can neutralize unstable oxygen-free radicals and other reactive oxygen species (ROS), and block free radical chain reactions [56,57,58]. Some components of TPs (such as Epigallocatechin gallate) can activate the Nrf2 antioxidant signaling pathway in hepatocytes, directly inducing the gene expression and protein synthesis of SOD [59]. In our experiment, the SOD activities in the green tea groups were significantly higher than those in the control group, while there was no significant difference among the green tea groups. For large yellow croaker (*Larimichthys crocea*), the SOD activity was similar at the levels of 100, 200, and 500 mg kg^−1^ of TPs [60], and the results of the two experiments are similar. The similarity in SOD among different test groups might be because low doses of TPs can trigger the maximum induction capacity of the Nrf2 pathway, and high doses cannot further enhance the effect [61].

GSH can eliminate free radicals and alleviate oxidative stress, and its decline leads to an increase in oxidative stress in the body, which is harmful to growth [62,63]. The core function of CAT is to eliminate hydrogen peroxide catalyzed by SOD [64]. The decrease in CAT activity means a decrease in the decomposition rate of hydrogen peroxide (H_2_O_2_), generating a large amount of ROS, which is harmful to organisms [65]. In our study, the FER and SGR in the G8000 group were also significantly the lowest, and they had a corresponding relationship with the significantly lowest CAT and GSH contents in the G8000 group. Conversely, the CAT activity in the G1000 group was significantly higher than that in the control group, with the highest FER corresponding to G1000. The above research indicates that the enhancement of antioxidant capacity in fish is positively correlated with FER improvement. Fish in aquaculture are exposed to stress, which leads to the accumulation of ROS in their bodies and causes oxidative damage; the body needs to consume a large amount of nutrients to repair this damage; and the improvement of antioxidant capacity (e.g., via enhancement of antioxidant enzyme activities such as SOD, CAT, and GSH-Px, and reduction in oxidative products such as MDA) can neutralize ROS and inhibit lipid peroxidation, thereby reducing the degree of oxidative damage and lowering the nutritional requirements for repairing oxidative damage, leading to more nutrients being used for growth [66]. Additionally, the improvement in antioxidant capacity can activate certain immune pathways, increase immune cell activity, and reduce the nutritional loss caused by diseases, enabling more feed nutrients to be used for growth and thus improving the feed conversion rate [67]. CAT can catalyze the decomposition of hydrogen peroxide (H_2_O_2_) into water and oxygen, reducing the cell damage caused by ROS [68]. Feeding grass carp dietary TPs at 0, 200, 400, 600, and 800 mg kg^−1^ [69], and Nile tilapia at 0, 83, 166, 333, and 666 mg kg^−1^ [70], the CAT contents in both species increased initially and then decreased, which is similar to the trend of CAT changes in our experiment, demonstrating a hormesis effect where low doses stimulate and high doses inhibit [71]. As an exogenous antioxidant, low-dose TPs can clear (or partially clear) excessive ROS in the fish body, rapidly reduce cell damage caused by ROS, activate antioxidant-related signaling pathways, promote the gene expression and protein synthesis of the CAT enzyme, and increase CAT activity [71]. Excessive doses of TPs can produce a large amount of phenoxyl radicals in the fish body, triggering oxidative stress. Excessive ROS will directly attack the CAT protein structure, reducing CAT catalytic activity; at the same time, continuous high oxidative pressure may damage the DNA or protein synthesis system of liver cells, reducing the production of CAT enzyme [72].

### 4.3. Effects on Intestinal Flora

In this study, the dominant bacterial communities in both the green tea group and the control group were Proteobacteria, Firmicutes, Bacteroides, etc., which were similar to previous research results in grass carp [73]. Green tea can inhibit the growth of harmful bacteria, and TPs play an important role in this process [74]. After feeding on 400 mg kg^−1^ TPs (supplemented in the diet), Crucian Carp HCC2 subjected to chronic overcrowding stress partially recovered intestinal Firmicutes abundance, while the abundance of stress-related pathogenic bacteria significantly decreased [12]. After feeding on 300 mg kg^−1^ TPs (supplemented in the diet), the diversity of intestinal flora and the abundance of probiotics was significantly increased in hybrid sturgeon (*Acipenser baerii* ♀ × *A. schrenckii* ♂), while harmful bacteria such as *Clostridium* significantly decreased; consequently, the immunity of the intestines was enhanced [14]. TPs can bind to the nucleic acids and enzymes of microorganisms, inhibit DNA replication, transcription, and the activity of certain metabolic enzymes, thereby suppressing the growth and reproduction of harmful bacteria [74]. Catechin compounds in TPs can combine with lipids and proteins on the cell membranes of harmful bacteria, change the permeability of the membranes, cause cytoplasmic leakage and death, and thus have a significant inhibitory effect on harmful bacteria [75]. The reduction in harmful bacteria lowers the contents of toxic substances such as endotoxins, ammonia, and indole in the intestinal tract, reducing the irritation to the intestinal mucosa and the inflammatory response [76]. *Gammaproteobacteria Incertae Sedis* contains many harmful bacteria, causing an imbalance in the host’s intestinal flora [77], and most species of *Bacteroides* can cause abdominal infections [78]. In our study, the decline in the abundance of these two types of flora indicates beneficial effects for the intestinal health of fish.

Green tea regulates intestinal metabolites, making the intestinal pH slightly acidic. Meanwhile, TPs have antioxidant effects, alleviating intestinal oxidative stress and reducing free radical damage to beneficial bacteria—all of which promote the growth of beneficial bacteria [12]. Additionally, reduced harmful bacteria increase intestinal attachment sites and nutrient resources, further favoring beneficial bacterial proliferation [14]. In this study, the abundance of *Peptostreptococcaceae* sp. significantly increased, which can promote food digestion, synthesize vitamins, occupy intestinal ecological niches, and produce antibacterial substances to resist exogenous pathogens, thereby maintaining intestinal health [79,80]. *Faecalibacterium prausnitzii(s)* [81], *Roseburia* [82], and *Lachnospiraceae bacterium G11* [83] are important producers of butyrate in the intestine. Veillonellales Selenomonadales can participate in lactic acid fermentation to form propionic acid and acetate [84], and Prevotellaceae can promote the synthesis of short-chain fatty acids [85]. In this study, the abundance of the above flora, closely related to the synthesis of short-chain fatty acids, increased significantly. Short-chain fatty acids are very important for maintaining the normal physiological functions of the intestinal tract. They can enhance the barrier function of the intestinal tract, prevent (or reduce) bacteria and toxins in the intestinal tract from entering the bloodstream, and lower the risk of inflammatory responses and various diseases [86]. Therefore, the relative abundance of these flora significantly increased, which can enhance the immunity of the intestinal flora [86].

Enhanced immunity is highly beneficial for aquaculture, as it reduces production costs and improves the aquatic environment [87]. For instance, dietary *Clostridium butyricum* supplementation in *Oreochromis niloticus* increased intestinal flora diversity, enhanced the abundance of beneficial bacteria (e.g., *Bacillus*), and reduced the levels of pathogenic bacteria (e.g., *Aeromonas*). After 56 days of culture, challenge with *Streptococcus agalactiae* resulted in significantly lower cumulative mortality and enhanced disease resistance, indirectly reducing the need for antibacterial agents in aquaculture [87]. However, green tea hinders the digestion of carbohydrates. In this study, there were significant decreases in Rubritaleaceae, *Lutibacter*, *Lutibacter yonseiensis*, Chloroflexi, Chloroflexia, and Bacteroidaceae—all of which are effective at decomposing carbohydrates [73,88,89,90]. The decline in these intestinal flora may have reduced the intestinal ability to break down carbohydrates. In the experiment, we did not investigate the effects of green tea on intestinal flora in other treatment groups. Thus, the correlation between green tea dosage and intestinal flora alterations could not be clarified, which is a limitation of this research.

## 5. Conclusions

The study showed that under the experimental conditions employed, supplementation with 1000 mg kg^−1^ of green tea appeared to be optimal for promoting growth, antioxidant capacity, and intestinal immunity in grass carp.

## Figures and Tables

**Table 1:** The formulation and proximate composition analysis of the experimental diet.

Ingredients (%)	G0	G500	G1000	G2000	G4000	G8000
White fishmeal ^1^	15	15	15	15	15	15
Soybean meal ^2^	42	42	42	42	42	42
Rapeseed meal ^3^	10	10	10	10	10	10
Cottonseed meal ^4^	8	8	8	8	8	8
α-starch ^5^	4	4	4	4	4	4
Wheat meal ^6^	7.5	7.5	7.5	7.5	7.5	7.5
Soybean oil ^7^	3	3	3	3	3	3
Cellulose ^8^	7	6.95	6.90	6.80	6.60	6.20
Ca(H_2_PO_4_)_2_ ^9^	1.6	1.6	1.6	1.6	1.6	1.6
Choline Chloride ^9^	0.3	0.3	0.3	0.3	0.3	0.3
Vitamin C ^9^	0.1	0.1	0.1	0.1	0.1	0.1
Mineral premix ^10^	0.3	0.3	0.3	0.3	0.3	0.3
Vitamin premix ^11^	1.2	1.2	1.2	1.2	1.2	1.2
Green tea	0	0.05	0.10	0.20	0.40	0.80
Proximate composition						
Crude protein (%)	35.70	35.74	35.65	35.58	35.64	35.54
Crude lipid (%)	5.02	5.14	5.08	4.97	4.94	4.88
Ash (%)	6.45	6.38	6.49	6.36	6.41	6.50
Gross energy (MJ kg^−1^)	15.13	15.09	15.11	14.94	15.15	14.98

Note: ^1^ Guangdong Sinofeed Industry and Commerce Incorporated Co., Guangzhou, Guangdong, China (66.20% mdeprotein, 10.25% crude lipid). ^2^ Yihai Kerry (Harbin) Grain, Oil & Foodstuff Industry Co., Ltd., Harbin, Heilongjiang, China. ^3^ Bunge (Dongguan) Grain and Oil Co., Ltd., Dongguan, Guangdong, China. ^4^ Wujiaqu Taikun Plant Protein Co., Ltd., Wujiiaqu, Xingjiang, China. ^5^ Hefeng (Dezhou) Material Technology Co., Ltd., Dezhou, Shandong, China. ^6^ Anhui Sizhou Flour Co., Ltd., Sizhou, Anhui, China. ^7^ China Sino-grain Oils & Fats (Xinzheng) Co., Ltd., Xingzheng, Henan, China. ^8^ Anhui Wanjiang Red Cellulose Co., Ltd., Fuyang, Anhui, China. ^9^ Beijing Sunpu Biochemical and Technology Co., Ltd., Beijing, China. ^10^ Mineral premix (g 100 g^−1^ premix): NaCl, 1; MgSO_4_ 7H_2_O, 15; NaH_2_PO_4_ 2H_2_O, 25; KH_2_PO_4_, 32; Ca(H_2_PO_4_)_2_ H_2_O, 20; FeC_6_H_5_O_7_ 5H_2_O, 2.5; (CH_3_CHOHCOO)_2_Ca·5H_2_O, 3.5; ZnSO_4_ 7H_2_O, 0.353; MnSO_4_ 4H_2_O, 0.162; GuSO_4_ 5H_2_O, 0.031; CoCl_2_ 6H_2_O, 0.001; KIO_3_, 0.003; cellulose, 0.45. ^11^ Vitamin premix (g 100g^−1^ premix): vitamin A, 550 I.U.; vitamin D_3_, 100 I.U.; vitamin E, 5 I.U.; vitamin K, 1; choline, 55; niacin, 10; riboflavin, 2; pyridoxine, 2; thiamin, 2; D-calcium pantothenate, 5; biotin, 0.01; folic acid, 0.5; vitamin B_12_, 2; ascorbic acid, 10; inositol, 10.

**Table 2:** Effects of green tea on growth performance and body indices of grass carp (mean ± SD, *n* = 3).

Group	G0	G500	G1000	G2000	G4000	G8000
IBW (g)	7.46 ± 0.13	7.46 ± 0.02	7.44 ± 0.10	7.44 ± 0.10	7.39 ± 0.07	7.44 ± 0.08
FBW (g)	36.32 ± 2.27 ^a^	37.02 ± 2.79 ^a^	37.54 ± 2.52 ^a^	37.14 ± 2.87 ^a^	31.83 ± 2.61 ^b^	29.78 ± 1.57 ^b^
SGR (% d^−1^)	2.93 ± 0.15 ^a^	2.96 ± 0.15 ^a^	3.01 ± 0.13 ^a^	2.97 ± 0.17 ^a^	2.70 ± 0.16 ^ab^	2.57 ± 0.12 ^b^
FR (% bw d^−1^)	3.16 ± 0.05 ^a^	3.15 ± 0.10 ^a^	3.09 ± 0.08 ^a^	3.19 ± 0.07 ^a^	3.36 ± 0.03 ^b^	3.47 ± 0.04 ^c^
FER (%)	75.03 ± 1.13 ^a^	77.83 ± 3.24 ^ab^	78.64 ± 1.01 ^b^	75.55 ± 2.35 ^ab^	68.08 ± 2.32 ^c^	63.51 ± 1.39 ^c^
CF (g cm^−3^)	1.87 ± 0.04	1.81 ± 0.09	1.84 ± 0.01	1.83 ± 0.06	1.89 ± 0.05	1.90 ± 0.02
VSI (%)	11.27 ± 0.15	10.78 ± 0.31	11.18 ± 1.08	11.04 ± 0.33	10.93 ± 0.50	11.13 ± 0.34
HIS (%)	1.78 ± 0.05 ^a^	1.66 ± 0.04 ^ab^	1.54 ± 0.06 ^b^	1.62 ± 0.05 ^ab^	1.69 ± 0.03 ^ab^	1.79 ± 0.10 ^a^
MFI (%)	2.51 ± 0.30	2.44 ± 0.16	2.32 ± 0.38	2.32 ± 0.49	2.31 ± 0.32	2.64 ± 0.28

Means with different superscripts within the same row are significantly different at *p* < 0.05.

**Table 3:** Effect of green tea on whole body composition of grass carp (on wet weight basis) (mean ± SD, *n* = 3).

Group	Moisture (%)	Crude Protein (%)	Crude Lipid (%)	Ash (%)
G0	77.44 ± 0.48	16.73 ± 0.55	4.48 ± 0.59	2.51 ± 0.19
G500	78.36 ± 0.64	16.14 ± 0.41	4.51 ± 0.65	2.61 ± 0.04
G1000	77.97 ± 0.88	17.10 ± 1.08	4.60 ± 0.40	2.62 ± 0.35
G2000	78.06 ± 0.61	16.44 ± 0.40	4.85 ± 0.28	2.34 ± 0.15
G4000	77.82 ± 0.40	16.72 ± 0.84	4.49 ± 0.42	2.59 ± 0.05
G8000	77.32 ± 0.09	17.25 ± 0.95	4.82 ± 0.37	2.57 ± 0.08

**Table 4:** Effect of green tea on intestinal digestive enzyme activity of grass carp (mean ± SD, *n* = 3).

Group	Trypsin (U mg^−1^ Prot)	Lipase (U g^−1^ Prot)	Amylase (U mg^−1^ Prot)
G0	28.67 ± 1.11 ^a^	16.66 ± 3.04	88.81 ± 4.18 ^a^
G500	29.15 ± 1.67 ^ab^	16.76 ± 0.82	80.58 ± 5.95 ^ab^
G1000	31.01 ± 1.09 ^b^	16.86 ± 1.60	75.52 ± 4.03 ^b^
G2000	28.58 ± 0.94 ^ab^	16.64 ± 1.65	81.44 ± 3.05 ^ab^
G4000	27.64 ± 2.66 ^ab^	16.23 ± 2.21	80.16 ± 5.32 ^ab^
G8000	25.20 ± 2.37 ^a^	15.79 ± 1.37	60.12 ± 5.56 ^c^

Means with the different superscripts within the same row are significantly different at *p* < 0.05.

**Table 5:** Effects of green tea on metabolic enzyme activity and liver and muscle glycogen content of grass carp (mean ± SD, *n* = 3).

Group	G0	G500	G1000	G2000	G4000	G8000
HK (U g^−1^ prot)	4.45 ± 0.51 ^a^	5.82 ± 0.32 ^b^	6.22 ± 0.40 ^b^	5.74 ± 0.46 ^b^	5.43 ± 0.67 ^ab^	5.41 ± 0.48 ^ab^
PK (U g^−1^ prot)	10.52 ± 0.37 ^a^	11.87 ± 0.53 ^b^	12.70 ± 0.82 ^b^	11.88 ± 3.44 ^ab^	11.24 ± 2.36 ^ab^	11.85 ± 1.63 ^ab^
LDH (U mg^−1^ prot)	9.49 ± 1.07	9.04 ± 1.10	9.48 ± 0.60	8.25 ± 1.31	10.30 ± 0.93	10.08 ± 1.50
SDH (U mg^−1^ prot)	6.74 ± 0.37 ^a^	7.50 ± 0.93 ^a^	6.63 ± 0.45 ^a^	6.13 ± 0.53 ^ab^	6.23 ± 0.34 ^ab^	5.35 ± 0.29 ^b^
PEPCK (U g^−1^ prot)	43.69 ± 3.16 ^a^	42.50 ± 2.42 ^a^	43.56 ± 2.04 ^a^	41.80 ± 5.56 ^ab^	36.86 ± 2.21 ^bc^	32.89 ± 1.54 ^c^
G-6-Pase (U g^−1^ prot)	109.45 ± 5.12 ^a^	118.01 ± 10.87 ^a^	115.72 ± 7.04 ^a^	110.93 ± 6.08 ^a^	90.17 ± 6.56 ^b^	82.06 ± 5.78 ^b^
ALT (U g^−1^ prot)	24.36 ± 2.52 ^a^	24.88 ± 3.04 ^a^	24.22 ± 4.08 ^a^	24.06 ± 3.41 ^a^	22.22 ± 3.53 ^a^	15.09 ± 2.96 ^b^
AST (U g^−1^ prot)	24.00 ± 4.26 ^a^	27.07 ± 3.51 ^a^	26.25 ± 3.08 ^a^	24.05 ± 2.58 ^a^	26.06 ± 3.11 ^a^	37.92 ± 3.54 ^b^
LPL (U g^−1^ prot)	1.60 ± 0.07 ^a^	1.73 ± 0.04 ^ab^	1.86 ± 0.11 ^b^	1.86 ± 0.10 ^b^	1.63 ± 0.18 ^ab^	1.21 ± 0.07 ^c^
HL (U g^−1^ prot)	3.39 ± 0.11	3.48 ± 0.32	3.52 ± 0.41	3.11 ± 0.23	3.52 ± 0.36	3.26 ± 0.44
TL (U g^−1^ prot)	4.99 ± 0.12	5.21 ± 0.28	5.38 ± 0.31	4.97 ± 0.28	5.15 ± 0.53	4.47 ± 0.51
HG (mg g^−1^)	10.49 ± 1.29 ^a^	7.23 ± 0.71 ^b^	5.67 ± 0.39 ^c^	6.85 ± 0.92 ^bc^	6.92 ± 1.00 ^bc^	6.87 ± 0.57 ^bc^
MG (mg g^−1^)	0.30 ± 0.06	0.26 ± 0.02	0.27 ± 0.02	0.26 ± 0.05	0.25 ± 0.05	0.30 ± 0.02

Means with the different superscripts within the same row are significantly different at *p* < 0.05.

**Table 6:** Effects of green tea on liver antioxidant indices of grass carp (mean ± SD, *n* = 3).

	SOD (U mg^−1^ Prot)	MDA (nmol mg^−1^ Prot)	CAT (U mg^−1^ Prot)	T-AOC (U mg^−1^ Prot)	GSH (μmol g^−1^ Prot)	GPx (U mg^−1^ Prot)
G0	200.36 ± 7.54 ^a^	5.49 ± 0.56	30.02 ± 2.30 ^a^	3.19 ± 0.21 ^a^	25.24 ± 2.17 ^a^	1144.39 ± 127.21 ^a^
G500	246.05 ± 11 ^b^	5.37 ± 0.74	46.66 ± 6.13 ^b^	4.11 ± 0.13 ^b^	26.35 ± 3.09 ^a^	1480.74 ± 132.08 ^b^
G1000	247.51 ± 13.1 ^b^	6.40 ± 0.55	39.16 ± 4.01 ^b^	4.42 ± 0.24 ^b^	25.80 ± 1.34 ^a^	1527.40 ± 160.25 ^b^
G2000	247.56 ± 8.25 ^b^	6.54 ± 0.68	31.39 ± 4.21 ^a^	4.16 ± 0.3 ^b^	22.14 ± 3.47 ^ab^	1357.03 ± 102.56 ^ab^
G4000	255.07 ± 14.33 ^b^	5.00 ± 0.60	29.66 ± 1.49 ^a^	4.11 ± 0.34 ^b^	20.88 ± 2.49 ^ab^	1410.75 ± 156.25 ^ab^
G8000	249.83 ± 13.78 ^b^	4.96 ± 0.66	22.61 ± 3.67 ^c^	4.01 ± 0.26 ^b^	18.98 ± 2.53 ^b^	1517.55 ± 91.74 ^b^

Means with the different superscripts within the same column are significantly different at *p* < 0.05.

## Data Availability

The raw data supporting the conclusions of this article will be made available by the authors on request.

## Abbreviations

ALT	alanine aminotransferase
AST	aspartate aminotransferase
CAT	catalase
CF	condition factor
FER	feed efficiency ratio
FR	feeding rate
G-6-Pase	glucose-6-phosphatase
GPx	glutathione peroxidase
HG	hepatic glycogen
HIS	hepatosomatic index
HK	hexokinase
HL	hepatic lipase
LDA	linear discriminant analysis
LDH	lactic dehydrogenase
LEfSe	linear discriminant analysis effect size
LPL	lipoprotein lipase
MDA	malondialdehyde
MFI	mesenteric fat index
MG	muscle glycogen
OUT	operational taxonomic unit
PEPCK	phosphoenolpyruvate carboxykinase
PK	pyruvate kinase
ROS	reactive oxygen species
SDH	succinate dehydrogenase
SGR	specific growth rate
SOD	superoxide dismutase
T-AOC	total antioxidant capacity
TCA	tricarboxylic acid
TL	total lipase
TPs	tea polyphenols
VSI	viscerosomatic index
